# Acquisition Origin Matters: Clinical, Microbiological and Immunological Characteristics and Treatment Effects in Community- vs. Hospital-Acquired Septic Shock

**DOI:** 10.3390/antibiotics15020169

**Published:** 2026-02-05

**Authors:** Irene Coloretti, Martina Tosi, Emanuela Biagioni, Federica Morselli, Elena Munari, Jacopo Bertolini, Sara Ferrari, Marianna Meschiari, Erica Franceschini, Nathan D. Nielsen, Stefano Busani, Massimo Girardis

**Affiliations:** 1 Anesthesia and Intensive Care Medicine, University Hospital of Modena, University of Modena and Reggio Emilia, 41125 Modena, Italy; irene.coloretti@gmail.com (I.C.); tosimartina@gmail.com (M.T.); emanuela.biagioni@gmail.com (E.B.); fede.morselli@gmail.com (F.M.); elenamunari@hotmail.com (E.M.); 252510@studenti.unimore.it (J.B.); 253769@studenti.unimore.it (S.F.);; 2 Infectious Diseases Unit, University Hospital of Modena, University of Modena and Reggio Emilia, 41125 Modena, Italy; 3 Division of Pulmonary, Critical Care and Sleep Medicine, Department of Internal Medicine, University of New Mexico School of Medicine, Albuquerque, NM 87131, USA; nathan.nielsen@gmail.com; 4 Section of Transfusion Medicine and Therapeutic Pathology, Department of Pathology, University of New Mexico School of Medicine, Albuquerque, NM 87131, USA

**Keywords:** septic shock, community-acquired infection, hospital-acquired infection, multidrug-resistant organisms, immune response, personalised medicine

## Abstract

**Background**: Septic shock is a leading cause of mortality worldwide, with community-acquired (CA) and hospital-acquired (HA) infections representing distinct clinical entities. The differences in clinical characteristics, immune response profiles, and effects of sepsis treatments between CA and HA septic shock have not been fully elucidated. **Methods**: This retrospective cohort study included 726 adults with septic shock who were admitted to two ICUs at Modena University Hospital between January 2006 and September 2024. Patients were classified as having CA or HA septic shock based on the origin of the infection. Clinical, microbiological, and immunological data, treatments, and outcomes were analysed. Immune cell dynamics were assessed during the first week after the onset of the shock. Multivariable Cox regression models were used to identify predictors and the effects of treatment on ICU mortality. **Results**: Among 344 patients with CA and 382 with HA septic shock, those with CA had higher severity scores but lower ICU and in-hospital mortality. Patients with HA exhibited a higher prevalence of multidrug-resistant organisms and more comorbidities. Immunologically, CA survivors showed increasing lymphocyte counts over time, whereas HA survivors mainly demonstrated recovery in T helper cells. Therapeutic strategies were similar between groups; however, continuous renal replacement therapy was more frequent in patients with HA. Neither appropriate empiric antibiotics nor steroids or immunoglobulin therapy independently improved mortality in the multivariate analyses. **Conclusions**: CA and HA septic shock differ significantly in terms of clinical severity, microbiological aetiology, immune recovery patterns, and outcomes. The lack of mortality benefit from standard treatments highlights the need for personalised management strategies that integrate clinical, immunological, and microbiological data to optimise care in septic shock subpopulations.

## 1. Introduction

Septic shock remains associated with mortality rates exceeding 40% in numerous countries [1] despite substantial advancements in pathophysiological understanding and treatment improvements. This persistent high mortality rate is attributable to the complexity and significant heterogeneity of septic shock, which are influenced by factors such as the source and nature of the underlying infection, as well as patient characteristics, including age, comorbidities, immune status, and prior treatments [2,3]. Furthermore, variations in therapeutic interventions, including the timing and appropriateness of antimicrobial therapy, fluid resuscitation, and supportive care, contribute to the diverse clinical presentations and outcomes observed in patients with septic shock [4,5]. According to sepsis-3 definition [6], septic shock is a severe subset of sepsis characterised by profound circulatory and metabolic abnormalities, requiring vasopressors to maintain MAP ≥ 65 mmHg and a serum lactate level > 2 mmol/L despite adequate fluid resuscitation.

Based on the origin of infection, septic shock cases can be categorised as community-acquired (CA) and hospital-acquired (HA), each presenting distinct epidemiological patterns and clinical implications [7]. CA septic shock is often caused by a different spectrum of pathogens compared to HA septic shock, which is frequently caused by multidrug-resistant organisms, complicating treatment and contributing to worse outcomes [8]. Beyond pathogens, CA and HA septic shock may differ significantly in terms of patient characteristics, clinical presentation, and outcomes. Patients with nosocomial septic shock often have a higher burden of comorbidities, are older, and have prior exposure to healthcare environments, including previous hospitalisations or invasive procedures [7]. These factors contribute to a more complex clinical profile and may impair immune function, increasing the vulnerability to severe infections and complicating recovery [9]. In contrast, community-acquired infections typically affect patients with fewer healthcare exposures and potentially different baseline health statuses [2]. Understanding these differences is essential for optimising early diagnosis, guiding targeted therapeutic strategies, and improving patient prognosis.

Numerous studies elucidated the clinical and microbiological differences between nosocomial and community-acquired infections and sepsis [2,7,8,10,11]. Nevertheless, there is a notable lack of studies specifically examining septic shock within these categories, particularly regarding variations in immune response, risk of secondary infections, treatment effects, and outcomes specific to patients with septic shock based on the infection’s origin. This study sought to thoroughly characterise the clinical and immunological features, as well as treatment effects and outcomes, of patients with septic shock with community-acquired versus nosocomial infections.

## 2. Results

**Figure 1:**
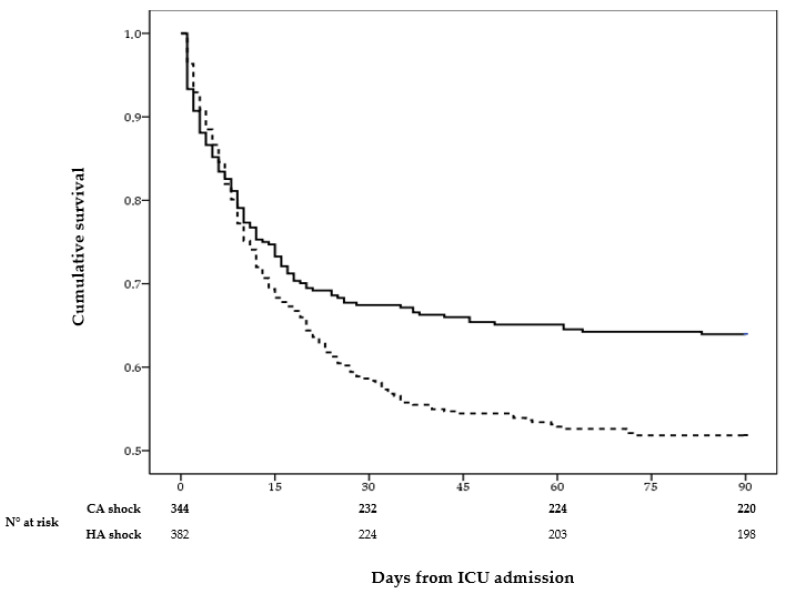
Kaplan–Meier curves for cumulative ICU survival in the comparison between CA and HA shock censored at 90 days. The solid line represents CA shock, and the dotted line represents HA shock. Log-rank test: *p* = 0.004.

Between January 2006 and September 2024, 726 patients with septic shock were included in the analysis, of whom 344 had CA and 382 had HA septic shock. At shock onset, patients with CA septic shock exhibited significantly higher SAPS II and SOFA scores than those with HA septic shock. In contrast, the HA group had a higher prevalence of pre-existing comorbidities and was more frequently affected by SARS-CoV-2 infection. The overall distribution of individual comorbidities at shock onset was similar between the two groups. Notably, 24.2% of the patients in the overall cohort had pre-existing immunosuppression (Table 1). Overall, the treatment strategies were largely comparable between the two groups; however, continuous renal replacement therapy (CRRT) was more frequently required in the HA group, showing a trend toward statistical significance (*p* = 0.059). Approximately 77% of patients received appropriate empirical antimicrobial therapy, with comparable rates in HA and CA septic shock cases (Table 1). ICU and in-hospital mortality were significantly lower among patients with CA shock compared with those with HA shock (*p* = 0.01 and *p* < 0.001, respectively) Consistently, ICU-free days were significantly higher in the CA group (Table 1). Kaplan–Meier ICU survival analysis over the 90-day follow-up period showed a lower cumulative survival probability (*p* = 0.004) in HA than in CA septic shock patients, with the divergence between the two groups becoming evident 10 days after shock onset and persisting throughout the observation period (Figure 1).

The most frequent primary site of infection was the lung (47%), with comparable proportions in the CA and HA shock groups. Overall, a microbiological pathogen was identified in 86.9% of the patients. Gram-negative bacteria were the predominant isolates, with *Escherichia coli* being the most frequently identified microorganism, accounting for 24.4% of all the isolates. *Pseudomonas aeruginosa* and *Enterococcus* spp. were more commonly isolated in patients with HA shock, whereas *Streptococcus* spp. were more frequently observed in CA shock. MDR organisms accounted for 47.8% of all isolates, with a significantly higher prevalence in the HA group than in the CA group (59.1% vs. 34.7%, respectively; *p* < 0.001). Carbapenem-resistant microorganisms were approximately twice as frequent in patients with HA shock. The incidence of secondary bacterial infections during ICU stay was slightly lower (*p* > 0.05) in the CA group than in the HA group (Table 2).

**Figure 2:**
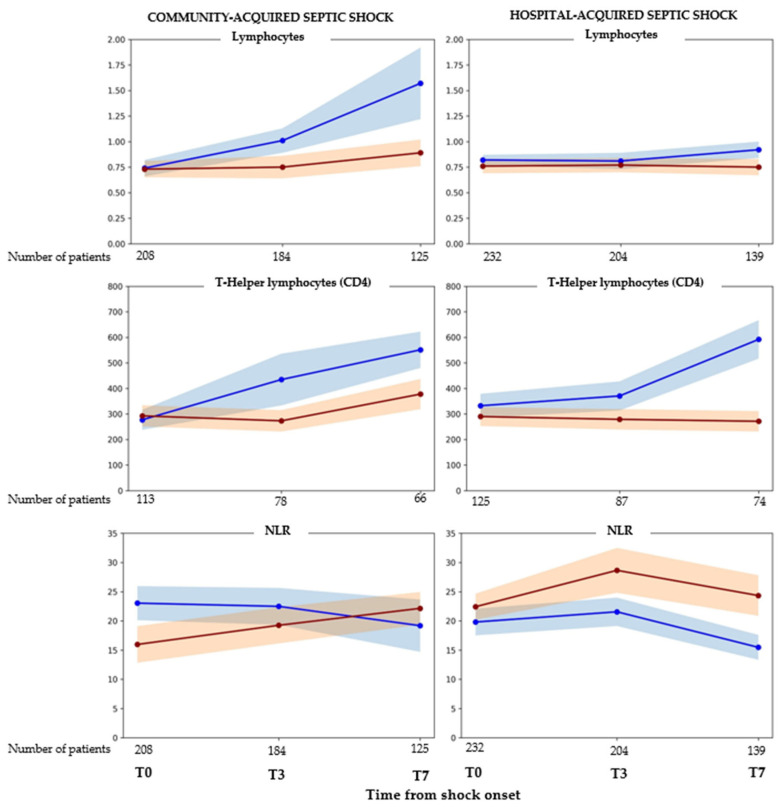
Temporal trends in immune cell populations and inflammatory markers according to acquisition setting and survival status at 30 days. Mean values with standard deviation (shaded bands) are shown at shock onset (T0), 3 (T3), and 7 (T7) days after shock onset. Panels depict community-acquired septic shock (**left** column) and healthcare-associated septic shock (**right** column). Blue line: ICU survivors; red line: ICU non-survivors.

At shock onset, patients with CA shock had significantly lower platelet and monocyte counts and higher procalcitonin levels than those with HA shock. Both groups demonstrated marked lymphopenia, with a median lymphocyte count of 0.64 (IQR 0.39–0.95) × 10^3^/mm^3^ in the overall cohort (Table 3). Analyses comparing CA and HA shock revealed distinct immunological patterns in the first week after shock development (Figure 2 and Figures S2–S9). In patients with CA shock, lymphocyte counts increased over time, with a significantly steeper increase in ICU survivors than in non-survivors, as indicated by a significant time-by-survival interaction (β = −0.31, *p* = 0.044). Temporal changes in T helper counts and neutrophil-to-lymphocyte ratio (NLR) were not significantly associated with survival, although a trend toward greater T helper recovery in survivors was observed (*p* = 0.056). In patients with HA shock, only T helper cells exhibited a survival-related temporal pattern, with a markedly greater recovery in ICU survivors than in non-survivors (β = −126.3, *p* = 0.001). Immunoglobulin levels did not show any difference in trend between survivors and non-survivors in the CA and HA groups (Figure S9). None of the additional immune cell subsets and immunoglobulin levels showed significant differences in temporal trajectories between survivors and non-survivors in the two groups (Supplemental Materials Figures S2–S8).

Multivariable Cox proportional hazards regression analysis identified per-year increase in age, SAPS II score, and liver cirrhosis as independent predictors of increased ICU mortality censored at 90 days (*p* < 0.05) in the subgroup of patients with CA shock (Table 4). Although there were signals for beneficial effects in the univariate analysis, appropriate empiric antibiotic and immunoglobulin therapies were not associated with improved mortality in the multivariate analysis. Among patients with HA shock, multivariable Cox regression analysis demonstrated that higher SAPS II scores and liver cirrhosis were independently associated with an increased risk of ICU mortality censored at 90 days (*p* < 0.05) (Table 4). None of the evaluated treatments showed any effect on mortality in the univariate or multivariate analyses. To assess the potential impact of temporal variations, we performed a sensitivity analysis by including distinct time intervals (e.g., 2006–2010, 2011–2016, 2017–2024) into the multivariable Cox proportional hazards regression model. This analysis did not identify any independent association between the time intervals and mortality, nor did it reveal any significant modification in the factors associated with mortality as observed in the primary analysis (see Supplementary Materials Figure S1).

## 3. Discussion

This comprehensive retrospective analysis, which included over 700 patients, identified key differences between community-acquired (CA) and hospital-acquired (HA) septic shock in terms of clinical and microbiological characteristics, immunological profiles, and treatment effects. To the best of our knowledge, this study represents the first instance of real-world data delineating the differences in immune profiles and treatment effects between CA and HA septic shock in patients admitted to the ICU. These insights may be crucial for developing personalised management strategies that address the distinct challenges posed by each type of infection within the context of septic shock.

Patients experiencing community-acquired (CA) septic shock exhibited higher severity scores, specifically SAPS II and SOFA, yet demonstrated significantly lower mortality rates both in the intensive care unit (ICU) and in-hospital settings compared to those with hospital-acquired (HA) septic shock. The observed differences may be attributed to host factors, including underlying comorbidities and immune status, as well as pathogen characteristics. Notably, HA septic shock was associated with a higher prevalence of multidrug-resistant (MDR) pathogens, such as carbapenem-resistant microorganisms, and a greater burden of comorbidities. When compared with existing literature, these clinical and microbiological distinctions between CA and HA infections are consistent with previous studies, which have reported a higher prevalence of MDR pathogens and poorer outcomes in HA sepsis due to resistant organisms and patient vulnerability [7,8].

The greater severity scores in CA septic shock contrast with other studies reporting higher severity scores in HA infections and sepsis [11]. This discrepancy may be attributed to the cohort of patients included in the various studies, which typically comprise individuals with infections or sepsis rather than exclusively those with septic shock, as in our study. Notably, elevated severity scores, in conjunction with reduced platelet and monocyte counts and increased procalcitonin levels, suggest a heightened inflammatory response in CA patients compared to that in HA patients. Higher levels of procalcitonin have been previously reported in patients with community-acquired pneumonia compared to those with ventilator pneumonia and were associated with the severity of illness [12].

Interestingly, although no differences between the HA and CA groups were observed at baseline (shock onset) in immune cell distribution and immunoglobulin levels, the analysis of changes over time showed some significant differences. Patients with CA shock exhibited an increase in total T lymphocyte and T-helper lymphocyte counts over time among survivors, while patients with HA shock showed survival-associated recovery primarily in T helper cells. Furthermore, the total lymphocyte counts on days 3 and 7 were higher in CA survivors than in HA survivors, suggesting a distinct trajectory in immune reconstitution, with potentially prolonged dysregulation in HA patients. Temporal changes in the NLR and other immune cell subsets in patients with HA and CA septic shock have not been previously described. A prospective analysis involving approximately 500 patients with sepsis revealed that the extent and pattern of immune cell dysfunction varied according to the type and source of infection, with hospital-acquired (HA) infections exhibiting more pronounced immunosuppression [13]. However, this analysis was conducted solely within the first 24 h following sepsis diagnosis. Another study comparing the systemic host response in critically ill patients with community-acquired (CA) and HA pneumonia focused only on the time of ICU admission [14]. The study revealed largely comparable outcomes concerning plasma biomarkers associated with inflammation, coagulation activation and endothelial cell activation. However, there was a slight indication of underexpression of a type I interferon gene signature in HA, suggesting more pronounced immune dysregulation in this population. Moreover, while there was no temporal difference in NLR between survivors and non-survivors across both cohorts, in patients with CA septic shock, the NLR remained elevated in non-survivors compared to survivors. Conversely, in patients with HA septic shock, NLR was lower in non-survivors than in survivors during the first three days. Numerous studies have shown that high NLR levels in sepsis or septic shock occurrence, as well as during the course of sepsis, are associated with an increased risk of mortality in sepsis [15,16,17].

The therapeutic strategies were largely uniform between the HA and CA groups. However, the HA group required continuous renal replacement therapy (CRRT) more frequently, indicating greater severity of organ dysfunction during their ICU stay. Despite some positive effects revealed in the univariate analysis, the multivariable analysis showed that none of the therapeutic strategies, including appropriate empiric antibiotics, steroids, and immunoglobulin therapy, independently influenced mortality in either group. The lack of significant treatment effects on mortality may be attributed to several key factors. First, the high percentage of appropriate antibiotic therapy administered in both groups likely minimised the differences in outcomes related to antimicrobial management. In addition, although antibiotic therapy was administered according to specific protocols that defined the timing, choice of molecule, and dosage, it is important to acknowledge that variations in these factors, particularly timing and dosage, could have impacted the effects on the outcome [18,19]. This is especially relevant when dealing with difficult-to-treat bacterial infections, where suboptimal timing or insufficient dosing may reduce the therapeutic effectiveness. Such influences might be more pronounced in the HA group, where resistant or more virulent pathogens are prevalent. Second, the use of steroids and immunoglobulins was guided by protocols based on the severity of clinical presentations, as indicated by guidelines [20] and consensus documents [21]. As previously demonstrated, this approach may not provide clear benefits to the general population [20]. Interestingly, the lack of beneficial effects of appropriate empiric antibiotic therapy and sepsis treatments on mortality has been previously reported in patients with MDR infections [22,23] and even in other populations at high risk, such as cirrhotic patients with septic shock [24], that was around 10% of the patients in our cohort. The lack of consistent benefits from standardised sepsis treatments depends on the profound heterogeneity, complexity, and dynamic nature of the host’s pathophysiological response to infection. Moreover, this variability is influenced by pre-existing patient conditions, such as cirrhosis or immunosuppression, and by the specific virulence and resistance profile of the infecting microorganism [5,25]. These considerations underscore the necessity of transitioning sepsis treatment from a uniform approach to a more personalised medicine strategy. This approach should be based on the integration of clinical, immunological, and microbiological data to determine the optimal strategy for specific sub-populations [26].

The strengths of this study are rooted in its comprehensive analysis of a substantial cohort of over 700 ICU patients diagnosed with septic shock. This study provides significant data that clearly distinguish the clinical, microbiological, and immunological characteristics of CA and HA septic shock. Temporal immunological profiling offers novel insights into immune recovery patterns related to survival, which have been scarcely addressed in prior research. However, limitations such as its retrospective, single-centre design may have introduced selection bias and limited generalisability. Moreover, SAPS II and SOFA scores were assessed only at the onset of shock and not longitudinally, which may represent a limitation when interpreting results. The long inclusion period spanning nearly two decades encompasses evolving sepsis definitions, treatment protocols, and pathogen resistance patterns, potentially confounding temporal trends despite reclassification efforts according to the Sepsis-3 criteria. The protocol for immunological monitoring was implemented in 2014, achieving a final compliance rate of approximately 50% among the patients included. In addition, the immunological assessments were limited to peripheral blood counts without functional immune profiling, which restricted mechanistic interpretations. Finally, the observational design and limited sample size ultimately constrain the capacity to draw causal inferences regarding treatment effects, particularly in relation to early and appropriate antibiotic therapy, which remains a fundamental aspect of sepsis management in septic shock. Despite adherence to specific protocols for antibiotic therapy, variations in treatment practices occurred over the 18-year period. These variations, including the introduction of new agents and evolving resistance patterns, could potentially limit the significance of the observed results.

## 4. Materials and Methods

This observational, retrospective cohort study was conducted in two multidisciplinary Intensive Care Units of Modena University Hospital. This retrospective study included consecutively admitted adult patients (≥18 years) with a diagnosis of septic shock between January 2006 and September 2024. Septic shock diagnosis was based on the Third International Consensus Definitions for Sepsis and Septic Shock (Sepsis-3) [6], even in the group admitted before 2016, whose clinical charts were revised. Patients with missing or uncertain data, those who were withheld life-sustaining treatments because they were too sick to benefit, and those not satisfying all the Sepsis-3 criteria were excluded. The study was approved by the Ethics Committee of Area Vasta Nord Emilia Romagna (approval number: 396/2020/OSS/AOUMO), which deemed informed consent unnecessary because of the retrospective design.

The study population was divided into two groups, community-acquired (CA) and hospital-acquired (HA) groups, based on the origin of infection leading to septic shock. In accordance with the criteria of the Centers for Disease Control and Prevention [27], an infection developing > 48 h after hospital admission or within 30 days after hospital discharge was defined as HA. Infections that occurred after hospital discharge are categorised as HA if they are attributable to the previous hospitalisation. An infection present on admission to the hospital or developing within 48 h or less from the time of admission was defined as CA.

Critically ill patients were considered ‘medical’ if admitted to the ICU for a medical problem and ‘surgical’ if admitted following scheduled or unscheduled surgery. The ICU admission criteria were based on the specific protocols of each unit.

A pathogen was classified as multidrug-resistant (MDR) if it demonstrated acquired non-susceptibility to at least one agent in three or more antimicrobial categories, according to standardised international definitions [28]. When available, antimicrobial susceptibility results were interpreted using breakpoints from the Clinical and Laboratory Standards Institute (CLSI) or the European Committee on Antimicrobial Susceptibility Testing (EUCAST) guidelines that were current at the time of isolate reporting (https://www.eucast.org/, accessed on 1 October 2023). Gram-negative bacteria were considered extended-spectrum beta-lactamase (ESBL)-producing pathogens if they belonged to the Enterobacteriaceae family and were resistant to more than one third-generation cephalosporin or aztreonam. Multidrug-resistant Pseudomonas species were defined as those resistant to at least three of the following antibiotics: Pseudomonas-active beta-lactams, carbapenems, aminoglycosides, and quinolones [29].

SARS-CoV-2 infection was defined as a positive result of real-time reverse transcriptase-polymerase chain reaction (RT-PCR) assay of nasopharyngeal swabs or lower-respiratory-tract specimens.

To facilitate longitudinal evaluation of immune variables, including lymphocyte subsets and immunoglobulins, a standardised immunomonitoring protocol has been routinely employed in our intensive care units for patients experiencing septic shock since 2014. Immune parameters are systematically assessed 72 h post onset and subsequently on a weekly basis throughout the duration of the ICU stay.

Demographics, comorbidities, medications and treatments, microbiological isolates, and laboratory tests were collected by reviewing electronic medical records. Empirical therapy was defined as appropriate when, in patients with a microbiological isolate, the administered treatment was active against the isolated microorganism according to the in vitro susceptibility testing.

Categorical variables are expressed as absolute numbers and percentages, and continuous variables as the median and interquartile range (IQR). For the comparison, the Chi-square test or Fisher’s exact test for categorical variables and the Mann–Whitney U-test for continuous variables were used. ICU survival censored at 90 days was estimated using the Kaplan–Meier method, and survival curves were compared between community-acquired and hospital-acquired septic shock using the log-rank test. The association between different variables and ICU mortality censored at day 90 was estimated using a multivariable Cox proportional hazards regression model, including all variables resulting in *p*-value < 0.2 in the unadjusted analysis, and variables deemed clinically relevant were forced into the model. Longitudinal differences in immunological and inflammatory parameters according to survival status were evaluated using linear mixed-effects models. Models included time, survival status, and their interaction as fixed effects, with subjects as random effects to account for repeated measures. The time-by-survival interaction term was used to assess differences in temporal trajectories between survivors and non-survivors. We confined our analysis to immunological data collected during the first week (T0, T3, and T7) due to the scarcity and fragmentation of data beyond this period, which impedes any comprehensive analysis. All analyses were restricted to complete cases; no methods were applied to handle missing data. SPSS version 22.0 package (SPSS Inc., Chicago, IL, USA) was used for statistical analysis.

## 5. Conclusions

In conclusion, this study revealed substantial clinical, microbiological, and immunological distinctions between CA and HA septic shock, thereby emphasising the limitations of standardised treatment protocols (Box 1). These results highlight the necessity of personalised management strategies specifically tailored to distinct patient subpopulations and the immune trajectory patterns seen here could inform future interventional or precision-medicine trials. To enhance patient outcomes, it is essential to conduct interventional trials tailored to the specific immune trajectories of individual patients. Additionally, further prospective real-world studies that integrate comprehensive clinical, immunological, and microbiological data are vital for optimising therapeutic interventions.

Box 1 **Summary of the differences between community acquired (CA) and hospital acquired (HA) septic shock.**

**KEY DIFFERENCES BETWEEN COMMUNITY-ACQUIRED AND HOSPITAL-ACQUIRED SEPTIC SHOCK:**

**Patient characteristics and microbiology:**

– CA patients had higher severity scores (SAPS II, SOFA) at shock onset.
– HA patients had more pre-existing comorbidities, including liver cirrhosis and immunosuppression.
– Surgical patients were more frequent in the HA group.
– HA septic shock showed higher prevalence multidrug-resistant organisms (MDROs).

**Immune response:**

– In CA patients, procalcitonin levels were higher, and platelet and monocyte counts were lower, indicating a more intense inflammatory response.
– CA survivors showed increasing total lymphocyte and T helper lymphocyte counts over the first week.
– HA patients exhibited prolonged immune dysregulation and less broad lymphocyte recovery.

**Treatment and organ support:**

– Invasive mechanical ventilation, empirical antibiotic therapy appropriateness, steroids and immunoglobulin use were similar across groups.
– HA patients required more frequently renal replacement therapy.

**Clinical outcomes:**

– HA patients exhibited significantly higher ICU and hospital mortality rates with survival curves diverged after 10 days.
– Slightly higher incidence of secondary bacterial infections in HA group.

**Predictors of mortality:**

– Age, SAPS II score, and liver cirrhosis independently predicted mortality.
– No treatment modality independently improved mortality in either group.

## Figures and Tables

**Table 1:** Description of the population characteristics at shock onset, treatments and outcomes in the comparison between CA and HA septic shock.

	All Population (n = 726)	CA Septic Shock (n = 344)	HA Septic Shock (n = 382)	*p* Value
**Age (year; median, IQR)**	70 (59–77)	70 (59–78)	69 (60–76)	0.339
**Sex, male (n, %)**	463 (63.8)	214 (62.2)	249 (65.2)	0.693
**SAPS II score (median, IQR)**	52 (41–67)	54 (44–69)	50 (39–66)	0.008
**SOFA score (median, IQR)**	10 (7–12)	10 (8–13)	9 (6–12)	0.003
**Comorbidities (n,%)**	577 (79.7)	253 (73.8)	324 (85.0)	<0.001
Chronic obstructive pulmonary disease (n,%)	88 (12.1)	47 (13.7)	41 (10.8)	0.226
Hearth_failure (n,%)	123 (17.0)	62 (18.1)	61 (16.1)	0.459
Diabetes (n,%)	135 (18.7)	72 (21.1)	63 (16.6)	0.124
Malignancy (n,%)	283 (38.9)	128 (37.2)	155 (40.6)	0.701
Pre-existing immune-suppression (n,%)	161 (24.2)	69 (22.2)	92 (25.9)	0.262
Liver cirrhosis (n,%)	85 (11.7)	37 (10.8)	48 (12.6)	0.441
SARS-CoV-2 infection (n,%)	118 (16.3)	31 (9.0)	87 (22.8)	<0.001
**Surgical patients (n,%)**	252 (35.0)	106 (31.1)	146 (38.6)	0.034
**Invasive mechanical ventilation (n,%)**	614 (84.6)	283 (82.3)	331 (86.6)	0.150
**Continuous renal replacement therapy (n,%)**	255 (35.1)	109 (31.7)	146 (38.2)	0.059
**Steroid therapy (n,%)**	505 (69.6)	227 (66.0)	278 (72.8)	0.654
**IgGAM therapy (n,%)**	363 (50.0)	181 (52.6)	182 (47.6)	0.181
**Appropriate antimicrobial therapy (n,%)**	489/631 (77.4)	226/291 (77.7)	263/340 (77.2)	0.896
**ICU mortality (n,%)**	312 (43.0)	125 (36.3)	187 (49.0)	0.001
**ICU-free days (median, IQR)**	0 (0–23)	15 (0–25)	0 (0–21)	<0.001
**Hospital mortality (n,%)**	408 (56.2)	164 (47.7)	244 (63.9)	<0.001

SAPS II score: Simplified Acute Physiology Score II; SOFA score: Sequential Organ Failure Assessment score; IgGAM: Immunoglobulins G, A, and M; ICU: Intensive Care Unit.

**Table 2:** Microbiological characteristics in the comparison between CA and HA septic shock.

	All Population (n = 726)	CA Septic Shock (n = 344)	HA Septic Shock (n = 382)	*p* Value
**Primary site of infection**				
Lung (n,%)	341 (47.0)	152 (44.2)	189 (49.5)	0.154
Abdomen (n,%)	177 (24.4)	76 (22.1)	101 (26.4)	0.279
BSI (n,%)	75 (10.3)	38 (11.0)	37 (9.7)	0.548
Other (n,%)	109 (15.0)	67 (19.5)	42 (11.0)	**0.001**
Unknown (n,%)	24 (3.3)	11 (3.2)	13 (3.4)	0.877
**Patients with microbial isolate (n,%)**	631 (86.9)	291 (84.6)	340 (89.0)	0.078
**Microorganisms isolated (n,%)**				
*Escherichia coli*	154 (24.4)	79 (27.1)	75 (22.1)	0.273
*Pseudomonas aeruginosa*	78 (12.4)	25 (8.6)	53 (15.6)	**0.004**
*Klebsiella pneumoniae*	70 (11.1)	32 (11.0)	38 (11.2)	0.769
*Acinetobacter baumanni*	26 (4.1)	14 (4.8)	12 (3.5)	0.501
Other Enterobacterales	53 (8.4)	20 (6.9)	33 (9.7)	0.144
*Stafilococcus aureus*	59 (9.4)	29 (10.0)	30 (8.8)	0.776
*Staphylococcus* spp.	21 (3.3)	8 (2.7)	13 (3.8)	0.387
*Enterococcus* spp.	43 (6.8)	13 (4.5)	30 (8.8)	**0.020**
*Streptococcus* spp.	36 (5.7)	29 (10.0)	7 (2.1)	**<0.001**
other	91 (14.4)	42 (14.4)	49 (14.4)	0.802
**Multi-drug resistant micro-organisms (n,%)**	302 (47.8)	101 (34.7)	201 (59.1)	**<0.001**
**Carbapenemase-producing micro-organisms (n,%)**	63 (10.0)	18 (6.2)	45 (13.2)	**<0.001**
**Secondary bacterial infection (n,%)**	49 (6.7)	19 (5.5)	30 (7.9)	0.077
**Time to secondary infection (median, IQR)**	12 (6–18)	13 (8–20)	**12 (6–17)**	**0.319**

BSI: Blood-stream infection; spp.: species.

**Table 3:** Laboratory data at shock onset in the comparison between CA and HA septic shock.

	All Population (n = 726)	CA Septic Shock (n = 344)	HA Septic Shock (n = 382)	*p* Value
**Platelets (×10^3^/mm^3^; median, IQR)**	176 (97–247)	148 (84–217)	186 (108–261)	**0.028**
**Creatinine (mg/dL; median, IQR)**	2.1 (1.4–3.3)	2.2 (1.6–3.3)	2.0 (1.3–3.1)	0.131
**Bilirubin (mg/dL; median, IQR)**	1.5 (0.8–3.5)	1.4 (0.8–3.1)	1.6 (0.9–4.2)	0.500
**PaO_2_/FiO_2_ (mmHg; median, IQR)**	206.3 (168.3–225.7)	209.5 (168.3–219.7)	205.0 (175.9–230.3)	0.775
**Lactate (mM/L; median, IQR)**	4.2 (2.2–6.4)	4.4 (2.1–7.5)	4.0 (2.4–6.1)	0.449
**Procalcitonin (ng/mL; median, IQR)**	15.8 (1.7–66.5)	31.7 (6.2–100.0)	6.6 (0.7–34.3)	**<0.001**
**WBC (median, IQR)**	10.4 (5.5–20.1)	10.8 (4.8–18.5)	10.4 (6.0–20.3)	0.539
**Neutrophil count (×10^3^/mm^3^; median, IQR)**	9.6 (5.2–18.1)	9.5 (4.6–16.3)	10.3 (6.1–18.7)	0.161
**Lymphocyte count (×10^3^/mm^3^; median, IQR)**	0.64 (0.39–0.95)	0.58 (0.38–0.89)	0.68 (0.43–1.00)	0.175
**NLR (median, IQR)**	15.3 (8.1–27.2)	14.7 (7.0–25.1)	15.7 (8.3–27.4)	0.417
**Monocytes (×10^3^/mm^3^; median, IQR)**	0.41 (0.15–0.68)	0.30 (0.15–0.47)	0.50 (0.17–0.78)	**0.053**
**T lymphocytes** **(×10^3^/mm^3^; median, IQR)**	372 (226–588)	335 (244–572)	405 (212–627)	0.695
**T helper lymphocytes** **(×10^3^/mm^3^; median, IQR)**	225 (138–390)	225 (139–355)	233 (138–411)	0.767
**T cytotoxic lymphocytes (×10^3^/mm^3^; median, IQR)**	121 (63–205)	109 (68–160)	142 (52–250)	0.240
**Natural Killer cells** **(×10^3^/mm^3^; median, IQR)**	81 (38–135)	91 (54–157)	70 (32–127)	0.060
**B lymphocytes (×10^3^/mm^3^;median, IQR)**	128 (57–199)	126 (67–199)	128 (56–195)	0.702
**IgG (mg/dL; median, IQR)**	702 (488–901)	690 (519–933)	709 (451–865)	0.333
**IgM (mg/dL; median, IQR)**	61 (37–110)	60 (38–117)	62 (37–108)	0.759
**IgA (mg/dL; median, IQR)**	176 (110–277)	176 (100–271)	179 (123–281)	0.526

WBC: White Blood Cells; Ig: Immunoglobulin.

**Table 4:** Univariate and multivariate analysis of factors independently associated with ICU mortality censored at 90 days among patients with CA septic shock (n = 344) and HA septic shock (n = 382).

COMMUNITY-ACQUIRED SEPTIC SHOCK						
	Survived to ICU	Dead in ICU	Unadjusted HR (95% CI);	*p* Value	Adjusted HR (95% CI);	*p* Value
	n = 219	n = 125				
**Age (years; median, IQR)**	70 (58–78)	72 (60–77)	**1.01 (0.99–1.03)**	**0.068**	**1.03 (1.00–1.05)**	**0.024**
**SAPSII score (median, IQR)**	50 (41–60)	67 (49–81)	**1.04 (1.03–1.05)**	**<0.001**	**1.02 (1.01–1.04)**	**0.004**
**Comorbidities (n,%)**	150 (68.8%)	103 (82.4%)	**1.91 (1.21–3.03)**	**0.006**	1.60 (0.90–2.84)	0.111
**Liver cirrhosis** **(n,%)**	11 (5.0%)	26 (20.8%)	**2.83 (1.84–4.37)**	**<0.001**	**2.83 (1.30–6.18)**	**0.009**
**Invasive Mechanical Ventilation (n,%)**	166 (76.1%)	117 (94.4%)	**1.70 (0.79–3.69)**	**0.178**	1.51 (0.62–3.72)	0.365
**Continuous renal replacement therapy (n,%)**	50 (22.9%)	59 (48.4%)	**1.31 (0.92–1.88)**	**0.136**	1.35 (0.82–2.24)	0.240
**Steroid therapy (n,%)**	144 (79.6%)	83 (90.2%)	**1.33 (0.67–2.65)**	**0.422**	1.53 (0.69–3.39)	0.292
**IgGAM therapy (n,%)**	125 (57.1%)	56 (44.8%)	**0.73 (0.52–1.04)**	**0.085**	0.79 (0.48–1.28)	0.336
**Appropriate empirical antimicrobial therapy (n,%)**	146 (84.9%)	80 (67.2%)	**0.63 (0.43–0.92)**	**0.018**	0.74 (0.42–1.32)	0.310
**HOSPITAL-ACQUIRED SEPTIC SHOCK**						
	**n = 195**	**n = 187**				
**Age (years; median, IQR)**	70 (61–76)	68 (59–75)	**1.00 (0.99–1.01)**	**0.790**	1.00 (0.98–1.01)	0.662
**SAPS II score (median, IQR)**	50 (38–64)	51 (39–70)	**1.02 (1.01–1.03)**	**<0.001**	**1.03 (1.02–1.04)**	**<0.001**
**Comorbidities (n,%)**	155 (79.5%)	169 (90.9%)	**1.99 (1.21–3.29)**	**0.007**	1.68 (0.89–3.16)	0.108
**Liver cirrhosis** **(n,%)**	15 (7.7)	33 (17.6)	**1.75 (1.20–2.55)**	**0.004**	**2.57 (1.39–4.75)**	**0.003**
**Invasive Mechanical Ventilation (n,%)**	159 (81.5%)	172 (94.0%)	**1.04 (0.56–1.93)**	**0.890**	0.78 (0.33–1.82)	0.562
**Continuous renal replacement therapy (n,%)**	53 (27.2%)	93 (51.4%)	**1.20 (0.90–1.61)**	**0.222**	0.98 (0.69–1.40)	0.907
**Steroid therapy (n,%)**	141 (80.1%)	137 (89.5%)	**1.54 (0.91–2.59)**	**0.105**	1.66 (0.88–3.13)	0.117
**IgGAM therapy (n,%)**	101 (51.8%)	81 (43.3%)	**0.86 (0.64–1.15)**	**0.302**	0.79 (0.54–1.15)	0.221
**Appropriate empirical antimicrobial therapy (n,%)**	135 (75.0%)	128 (80.0%)	**1.01 (0.68–1.50)**	**0.968**	0.88 (0.57–1.37)	0.573

SAPS II score: Simplified Acute Physiology Score II; IgGAM: Immunoglobulins G, A, and M.

## Data Availability

The data presented in this study are available on request from the corresponding author. The data are not publicly available due to privacy and ethical restrictions.

## Supplementary Materials

The following supporting information can be downloaded at: https://www.mdpi.com/article/10.3390/antibiotics15020169/s1, Figure S1: Comparison of patient numbers (bars), mortality rate (continuous line) across three time periods. MDR infections rates were 33.0% for the 1st period (2006–2010), 24.4% for the 2nd period (2011–206), 42.6% for the 3rd period (2017–2024). Figure S2: temporal trends in PCT according to acquisition setting and survival status at 30 days. Mean values with standard deviation (SD) are shown at T0, T3, and T7. Panels depict community-acquired septic shock (left column) and healthcare-associated septic shock (right column); Figure S3: temporal trends in WBC according to acquisition setting and survival status at 30 days. Mean values with standard deviation (SD) are shown at T0, T3, and T7. Panels depict community-acquired septic shock (left column) and healthcare-associated septic shock (right column); Figure S4: Temporal trends in monocytes according to acquisition setting and survival status at 30 days. Mean values with standard deviation (SD) are shown at T0, T3, and T7. Panels depict community-acquired septic shock (left column) and healthcare-associated septic shock (right column); Figure S5: temporal trends in T Lymphocytes (CD3) according to acquisition setting and survival status at 30 days. Mean values with standard deviation (SD) are shown at T0, T3, and T7. Panels depict community-acquired septic shock (left column) and healthcare-associated septic shock (right column); Figure S6: temporal trends in Cytotoxic T Lymphocytes (CD8) according to acquisition setting and survival status at 30 days. Mean values with standard deviation (SD) are shown at T0, T3, and T7. Panels depict community-acquired septic shock (left column) and healthcare-associated septic shock (right column); Figure S7: temporal trends in Natural Killer cells (CD16) according to acquisition setting and survival status at 30 days. Mean values with standard deviation (SD) are shown at T0, T3, and T7. Panels depict community-acquired septic shock (left column) and healthcare-associated septic shock (right column); Figure S8: temporal trends in B Lymphocytes (CD19) according to acquisition setting and survival status at 30 days. Mean values with standard deviation (SD) are shown at T0, T3, and T7. Panels depict community-acquired septic shock (left column) and healthcare-associated septic shock (right column); Figure S9: temporal trends in IgGAM according to acquisition setting and survival status at 30 days. Mean values with standard deviation (SD) are shown at T0, T3, and T7. Panels depict community-acquired septic shock (left column) and healthcare-associated septic shock (right column).
