# Supplementary material for: Acquisition Origin Matters: Clinical, Microbiological and Immunological Characteristics and Treatment Effects in Community- vs. Hospital-Acquired Septic Shock
Source: Antibiotics (Basel). 2026 Feb 5;15(2):169. doi: 10.3390/antibiotics15020169 (PMC12937308; doi:10.3390/antibiotics15020169)

Figure S1 – Comparison of patient numbers (bars), mortality rate (continuous line) across three time periods. MDR infections rates were 33,0% for the 1st period (2006-2010), 24,4% for the 2nd period (2011-206), 42,6% fot the 3rd period (2017-2024).

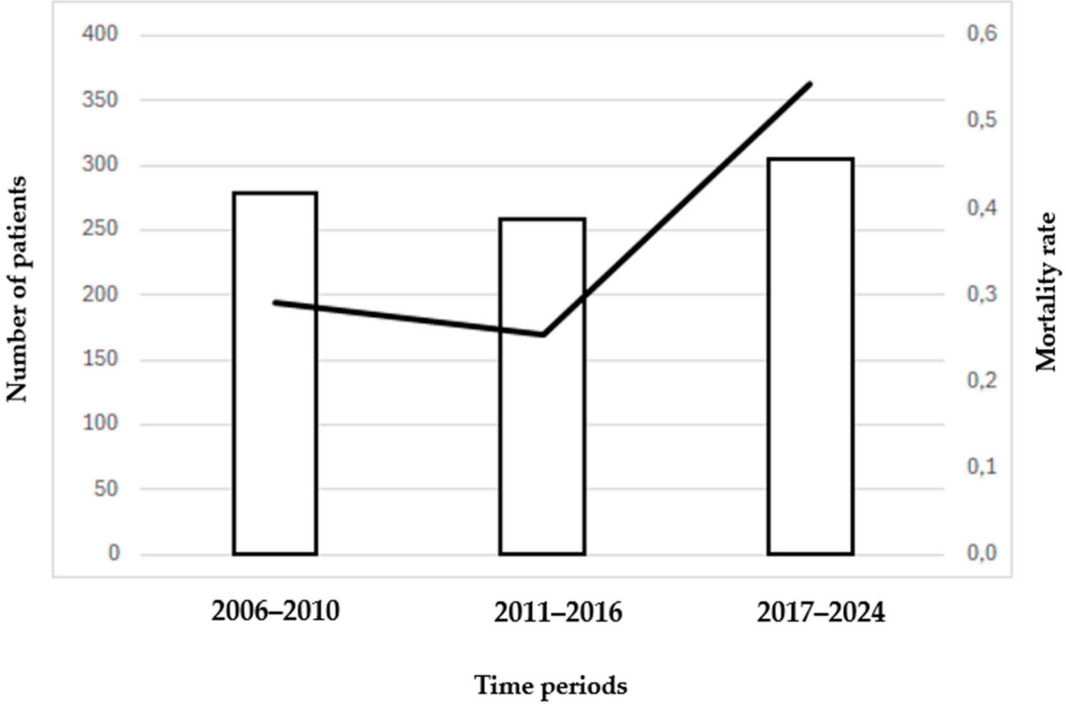

Figure S2 – Temporal trends in PCT according to acquisition setting and survival status at 30 days. Mean values with standard deviation (SD) are shown at T0, T3, and T7. Panels depict community-acquired septic shock (left column) and healthcare-associated septic shock (right column).

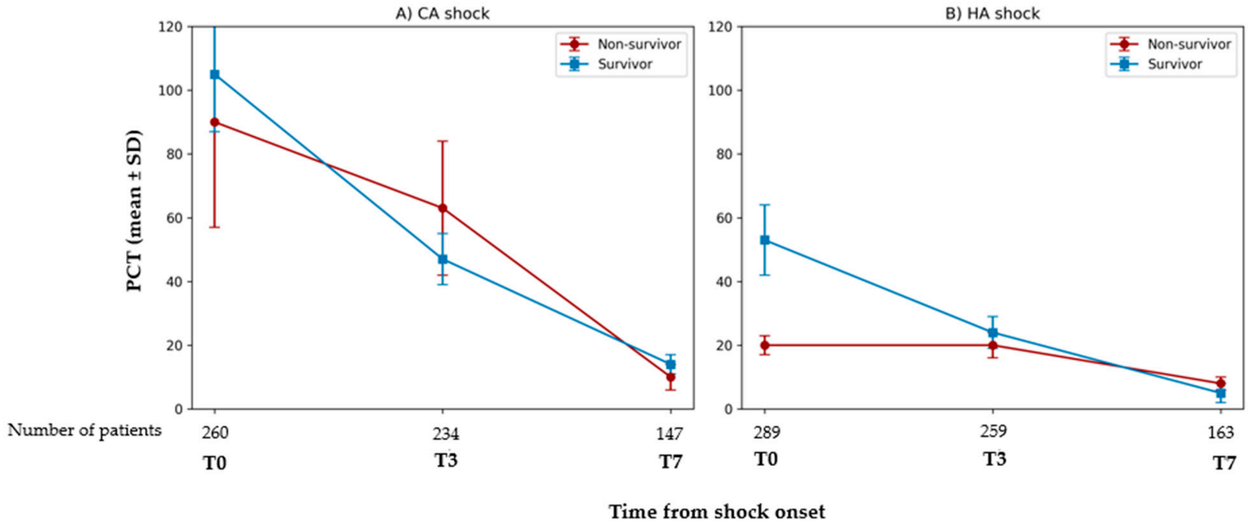

Figure S3 – Temporal trends in WBC according to acquisition setting and survival status at 30 days. Mean values with standard deviation (SD) are shown at T0, T3, and T7. Panels depict community-acquired septic shock (left column) and healthcare-associated septic shock (right column).

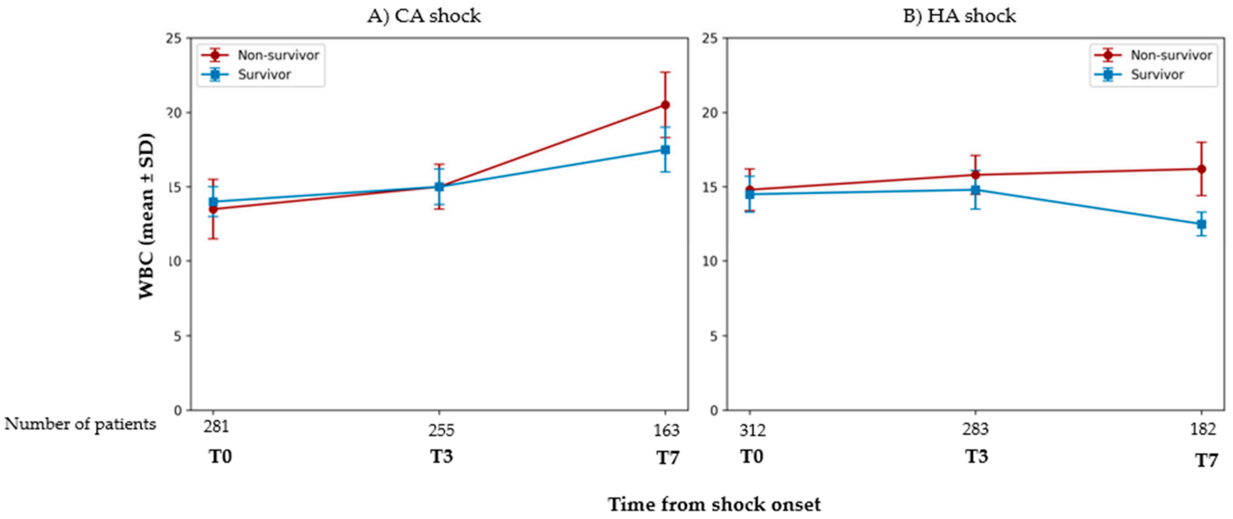

Figure S4 – Temporal trends in monocytes according to acquisition setting and survival status at 30 days. Mean values with standard deviation (SD) are shown at T0, T3, and T7. Panels depict community-acquired septic shock (left column) and healthcare-associated septic shock (right column).

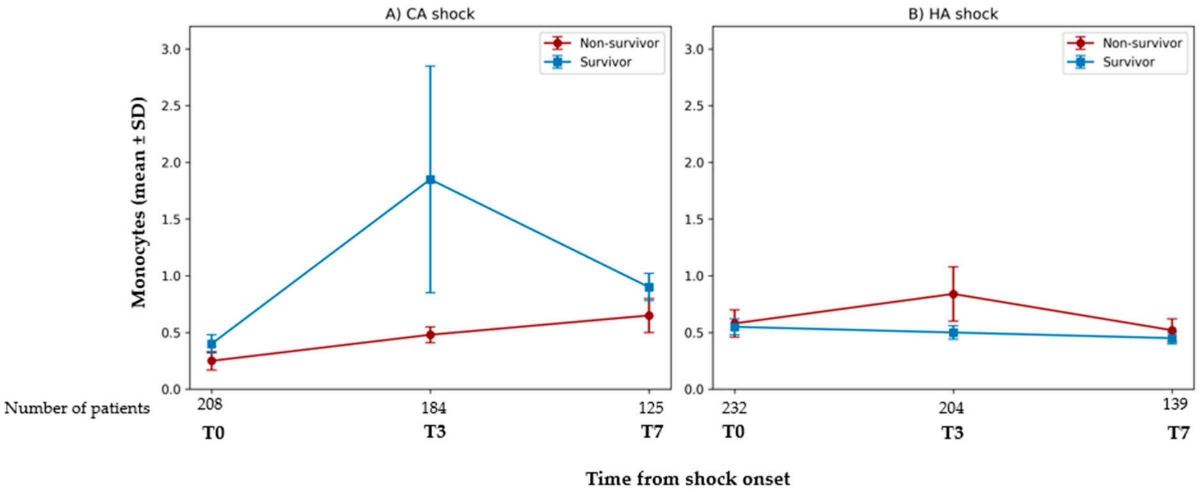

Figure S5 – Temporal trends in T Lymphocytes (CD3) according to acquisition setting and survival status at 30 days. Mean values with standard deviation (SD) are shown at T0, T3, and T7. Panels depict community-acquired septic shock (left column) and healthcare-associated septic shock (right column).

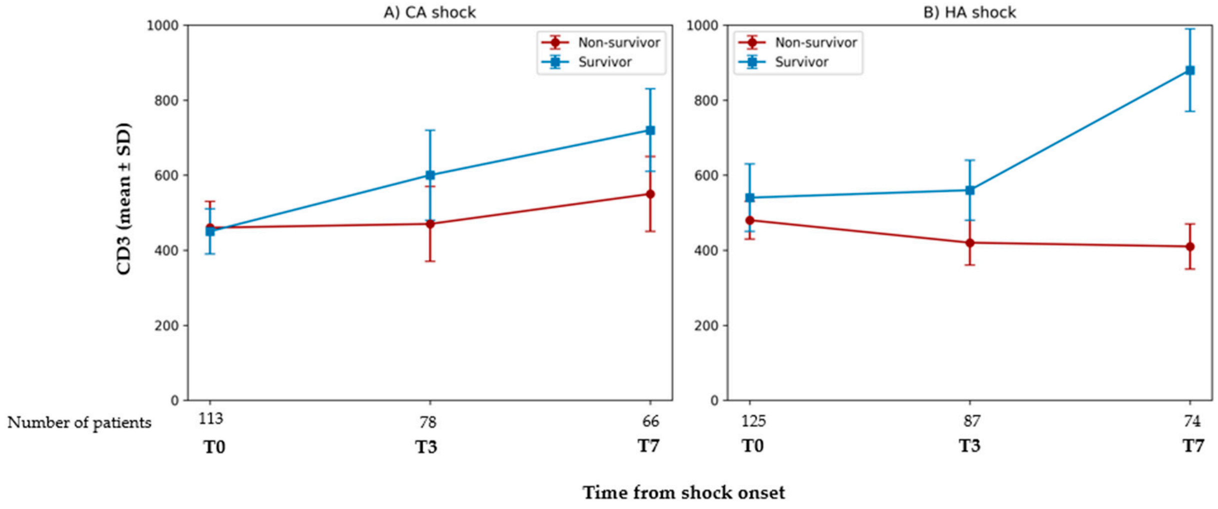

Figure S6 – Temporal trends in Cytotoxic T Lymphocytes (CD8) according to acquisition setting and survival status at 30 days. Mean values with standard deviation (SD) are shown at T0, T3, and T7. Panels depict community-acquired septic shock (left column) and healthcare-associated septic shock (right column).

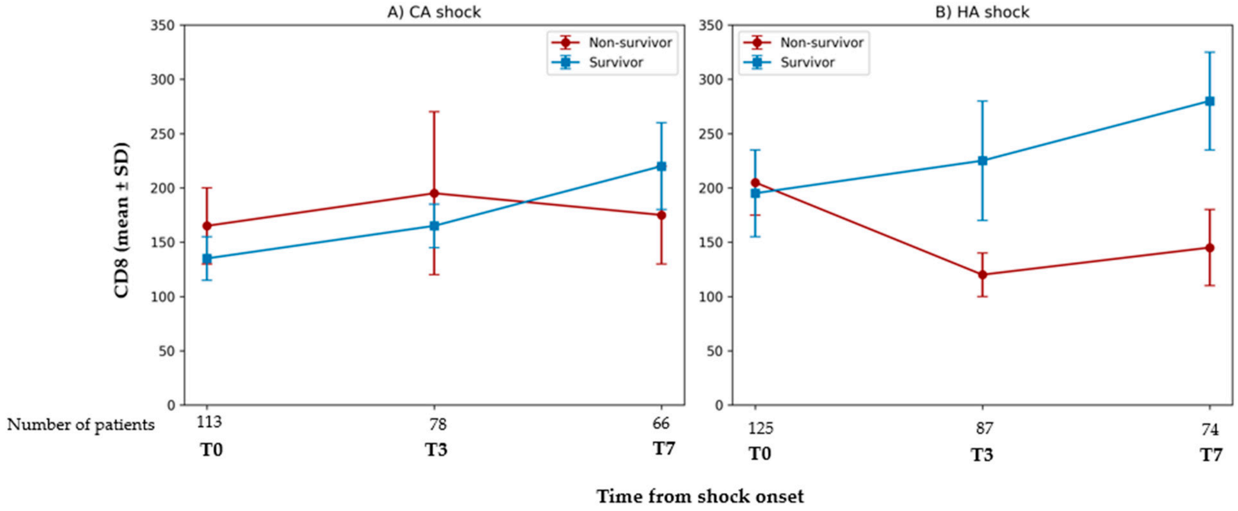

Figure S7 – Temporal trends in Natural Killer cells (CD16) according to acquisition setting and survival status at 30 days. Mean values with standard deviation (SD) are shown at T0, T3, and T7. Panels depict community-acquired septic shock (left column) and healthcare-associated septic shock (right column).

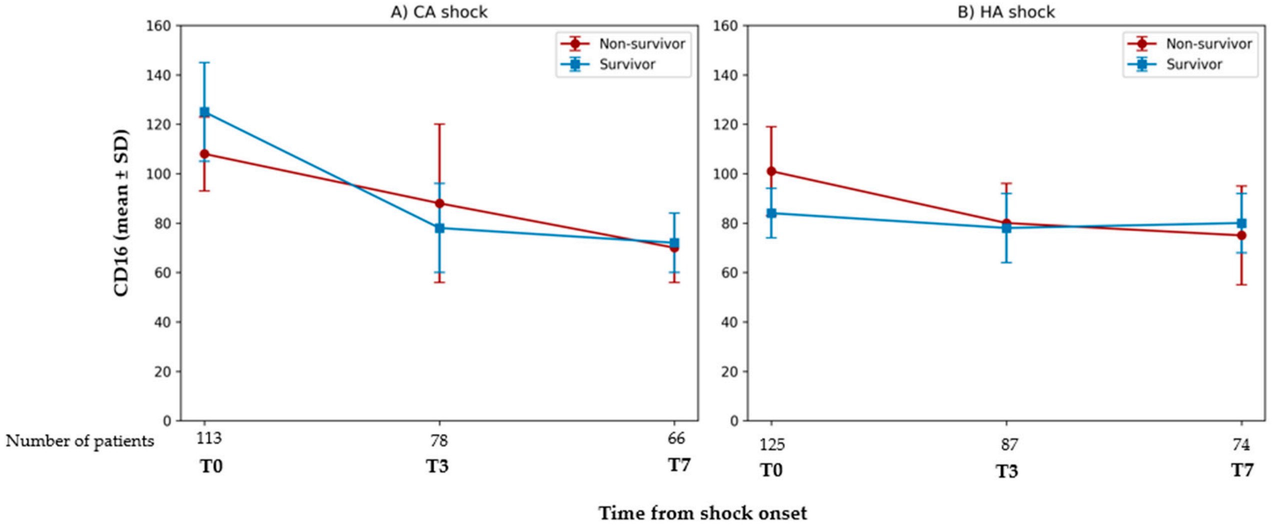

Figure S8– Temporal trends in B Lymphocytes (CD19) according to acquisition setting and survival status at 30 days. Mean values with standard deviation (SD) are shown at T0, T3, and T7. Panels depict community-acquired septic shock (left column) and healthcare-associated septic shock (right column).

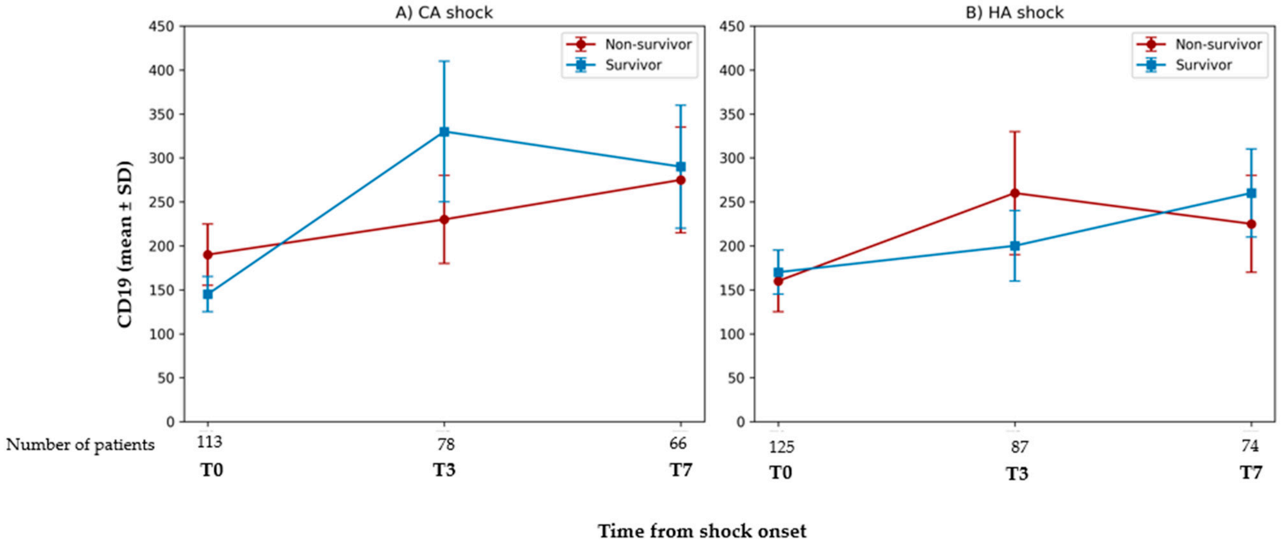

Figure S9– Temporal trends in IgGAM according to acquisition setting and survival status at 30 days. Mean values with standard deviation (SD) are shown at T0, T3, and T7. Panels depict community-acquired septic shock (left column) and healthcare-associated septic shock (right column).

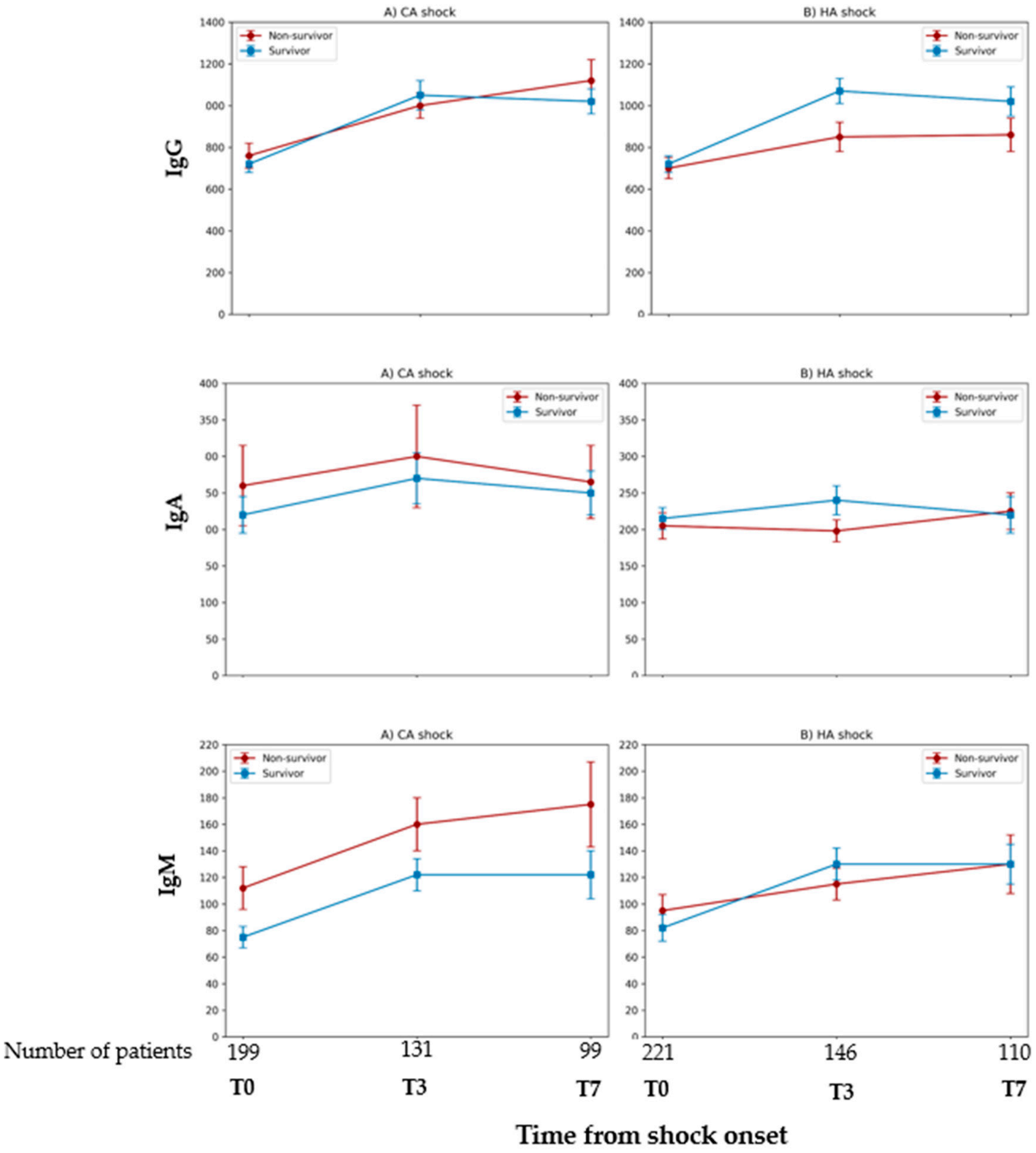

Supplement: Supplementary file 1 [file antibiotics-15-00169-s001.zip › antibiotics-4115494-supplementary.pdf]
